# Prognostic Value of MicroRNAs in Patients after Myocardial Infarction: A Substudy of PRAGUE-18

**DOI:** 10.1155/2019/2925019

**Published:** 2019-11-03

**Authors:** M. Hromádka, V. Černá, M. Pešta, A. Kučerová, J. Jarkovský, D. Rajdl, R. Rokyta, Z. Moťovská

**Affiliations:** ^1^Department of Cardiology, University Hospital and Faculty of Medicine of Charles University, Pilsen, Czech Republic; ^2^Department of Biology, Faculty of Medicine in Pilsen, Charles University, Czech Republic; ^3^Institute of Biostatistics and Analyses, Faculty of Medicine and the Faculty of Science, Masaryk University, Brno, Czech Republic; ^4^Department of Clinical Biochemistry and Hematology, University Hospital and Faculty of Medicine in Pilsen, Czech Republic; ^5^Cardiocentre, Third Faculty of Medicine, Charles University and University Hospital Kralovske Vinohrady, Prague, Czech Republic

## Abstract

**Background:**

The evaluation of the long-term risk of major adverse cardiovascular events and cardiac death in patients after acute myocardial infarction (AMI) is an established clinical process. Laboratory markers may significantly help with the risk stratification of these patients. Our objective was to find the relation of selected microRNAs to the standard markers of AMI and determine if these microRNAs can be used to identify patients at increased risk.

**Methods:**

Selected microRNAs (miR-1, miR-133a, and miR-499) were measured in a cohort of 122 patients from the PRAGUE-18 study (ticagrelor vs. prasugrel in AMI treated with primary percutaneous coronary intervention (pPCI)). The cohort was split into two subgroups: 116 patients who did not die (survivors) and 6 patients who died (nonsurvivors) during the 365-day period after AMI. Plasma levels of selected circulating miRNAs were then assessed in combination with high-sensitivity cardiac troponin T (hsTnT) and N-terminal probrain natriuretic peptide (NT-proBNP).

**Results:**

miR-1, miR-133a, and miR-499 correlated positively with NT-proBNP and hsTnT 24 hours after admission and negatively with left ventricular ejection fraction (LVEF). Both miR-1 and miR-133a positively correlated with hsTnT at admission. Median relative levels of all selected miRNAs were higher in the subgroup of nonsurvivors (*N* = 6) in comparison with survivors (*N* = 116), but the difference did not reach statistical significance. All patients in the nonsurvivor subgroup had miR-499 and NT-proBNP levels above the cut-off values (891.5 ng/L for NT-proBNP and 0.088 for miR-499), whereas in the survivor subgroup, only 28.4% of patients were above the cut-off values (*p* = 0.001).

**Conclusions:**

Statistically significant correlation was found between miR-1, miR-133a, and miR-499 and hsTnT, NT-proBNP, and LVEF. In addition, this analysis suggests that plasma levels of circulating miR-499 could contribute to the identification of patients at increased risk of death during the first year after AMI, especially when combined with NT-proBNP levels.

## 1. Introduction

The in-hospital mortality rate for acute myocardial infarction is low, due to efficient antiplatelet treatment and primary percutaneous coronary intervention (pPCI); unfortunately, the risk of cardiac death increases during the chronic phase of ischemic heart disease that follows.

Decreased left ventricular systolic function with left ventricular ejection fraction (LVEF) ≤ 35% and recurrent ventricular tachycardia or ventricular fibrillation, beyond the early phase of myocardial infarction, are connected with a poor prognosis and are a potential indication for cardioverter implantation [1].

Despite the clear benefit of these widely used predictors, they seem to be inadequate for identifying all patients at risk of sudden death, since it fails to identify about 50% of patients who die suddenly [2] after acute myocardial infarction (AMI). Some of the standard laboratory markers associated with the risk of sudden death can be used in combination with LVEF to improve the risk assessment process, but unfortunately, well-defined cut-off values are still not known.

Among factors that can be used for risk stratification after AMI, the following play an important role: elevated levels of troponin T or I (TnT or TnI) [3, 4] and a combination of (A) increased TnT and CRP plasma levels, (B) increased levels of N-terminal prohormone of brain natriuretic peptide (NT-proBNP) with LVEF < 40% [4–7], and (C) decreased clearance of creatinine (with a reduced LVEF) [8].

A promising group of new biomarkers, released from cells into circulation, is microRNAs (miRNAs), which are small noncoding RNA molecules, 20–22 nucleotides in length, involved in posttranscriptional regulation of gene expression. Mature miRNAs and Ago proteins (Argonaute proteins) form in the cytoplasm RISC complexes (RNA-induced silencing complexes) that interact with protein-coding mRNA molecules. This interaction usually leads to the inhibition of translation or directly to the degradation of mRNA molecules. One particular microRNA can regulate many genes (i.e., interacting with a variety of different protein-coding mRNAs), and one particular gene can be regulated by several different microRNAs. MicroRNAs can act directly within the cells where they are synthesized, or they can be exported, in complexes with proteins or in membrane-bound vesicles (exosomes or microvesicles), to other cells where they can also regulate gene expression. MicroRNAs are involved in the control of many processes in both healthy and infarcted myocardia, including proliferation, differentiation, apoptosis, repair, and revascularization [9]. Additionally, miRNA dysregulation has been strongly implicated in the destabilization and rupture of atherosclerotic plaques [10] as well as being involved in the process of myocardial recovery.

In cardiovascular diseases (CVD), the use of miRNAs as biomarkers for specific disease entities has been successfully investigated in numerous studies [11]. Nonetheless, it is not yet possible to use them in clinical practice [12]. miRNAs also have the potential for clinical use in CVD where protein biomarkers are not available.

More than 2500 mature miRNAs have been identified in humans. Four of them, miR-1, miR-133, miR-208a, and miR-499 have been found to be specific for the myocardium (or the myocardium and skeletal muscle) and are sometimes called “myomiRs” [13].

Many authors have shown that levels of circulating myomiRs increase significantly during the first few hours after the onset of myocardial infarction symptoms. After reaching a peak, myomiRs return to normal after a few hours or a few days [14, 15].

We decided to retrospectively measure the relative levels of circulating miR-1, miR-133a, miR-208a, and miR-499 in a well-described cohort of 122 patients with known one-year mortality, previously involved in the PRAGUE-18 study [16, 17]. The listed miRNAs were assessed alone and in combination with several standard markers in an effort to better characterize the nonsurvivor subgroup, with the goal of finding additional predictors of patients at increased risk of one-year cardiovascular death.

## 2. Material and Methods

### 2.1. Patients

The whole cohort of 122 patients was treated in the Department of Cardiology, University Hospital and Faculty of Medicine of Charles University, Pilsen, Czech Republic, which was one of the centers involved in phase IV of a multicenter, open-label, randomized, controlled clinical trial called the PRAGUE-18 study [16, 17].

The PRAGUE-18 study, which compared prasugrel and ticagrelor in the treatment of acute myocardial infarction, was the first randomized head-to-head comparison of these two active substances, with regard to efficacy and safety in patients after AMI undergoing pPCI. One of the outcomes was the combined endpoint of cardiovascular death, MI, or stroke within the first year. Prasugrel and ticagrelor had been similarly effective during the first year after AMI [16, 17]. Plasma samples from 122 patients in the study were used for this retrospective data analysis, where (I) levels of selected circulating microRNAs, (II) standard AMI biomarkers, and (III) LVEF were used to (A) look for correlations between miRNAs and standard AMI markers, (B) identify differences in biomarkers between survivors and nonsurvivors during the first year after AMI, and (C) better characterize the nonsurvivor subgroup relative to measures I, II, and III mentioned above.

### 2.2. Echocardiography

Two-dimensional, M-mode, and Doppler echocardiograms were acquired using an ultrasound system (Vivid 7, GE Medical Systems, Horton, Norway) using a 3.4 MHz multifrequency transducer. The systolic function of the left ventricle was determined according to the Simpson method from the apical 4-chamber view and the apical 2-chamber view (the biplane Simpson method).

### 2.3. Levels of Biomarkers

Data for the basic characteristics of all patients involved in the analysis were available from the PRAGUE-18 study. Levels of standard AMI biomarkers were known, including hsTnT, NT-proBNP, cystatin C, myoglobin, growth/differentiation factor 15 (GDF-15), and creatine kinase (CK) at patient admission and hsTnT also after 24 hours.

NT-proBNP was determined using the original analytical kits from Roche on a cobas® 8000 analyzer. NT-proBNP and high-sensitivity cardiac troponin were determined using the original analytical kits from Roche with the electrochemiluminiscence (ECLIA) principle on a cobas e602 analyzer. Imprecission of the hsTnT method on the 99th percentile was below 10% which is the required analytical performance specification. Growth/differentiation factor 15 (GDF-15) (RayBiotech, Norcross, USA) was determined using ELISA kits on a NEXgen Four ELISA reader (Adaltis, Rome, Italy).

Since hsTnT is the most frequently used standard biomarker of AMI and NT-proBNP is a sensitive marker of left ventricular dysfunction, we used them in combination with the potential new microRNA biomarkers, in subsequent analyses.

### 2.4. MicroRNA Analysis

#### 2.4.1. RNA Isolation

MicroRNA was isolated from plasma samples taken 24 hours after admission (all patients were already after pPCI at that time) and stored at −80°C. Total cell-free RNA was isolated from 200 *μ*L of plasma using miRNeasy Serum/Plasma Kits (miRNeasy Serum/Plasma Kit (50), Cat no./ID 217184; Qiagen, Hilden, Germany) according to the manufacturer's instructions. Total RNA was eluted in 14 *μ*L of ribonuclease-free water and stored at −80°C until further analyses. MicroRNA-39 (*C. elegans* miR-39) was used as the RNA spike-in control. A fixed volume of 1 *μ*L of this RNA eluate was used for each reverse transcription reaction.

#### 2.4.2. Quantitative Estimation of MicroRNA Expression

For reverse transcriptions and quantitative estimations of selected microRNAs using real-time PCR reactions, TaqMan® MicroRNA Assays and master mixes were used (catalogue number 4440887: hsa-miR-133a-3p—Assay ID 002246, hsa-miR-1-3p—Assay ID 002222, hsa-miR-499a-5p—Assay ID 001352, hsa-miR-208-3p—Assay ID 000511, and cel-miR-39-3p—Assay ID 000200; TaqMan Universal MMIX II: catalogue number 4440049; and TaqMan® MicroRNA RT Kit: catalogue number 4366597). A T100TM thermal cycler (Bio-Rad, California, United States) was used for reverse transcription. The reaction volume was 15 *μ*L. A fixed volume of 2.5 *μ*L from this RT reaction was used into each real-time PCR reaction. Due to either too high or absent Ct values, levels of miR-208a could not be quantified and evaluated.

#### 2.4.3. Processing of Real-Time PCR Data

Samples were assessed in technical duplicate. The Ct values were corrected using calibrators to eliminate differences between individual runs of the Stratagene Mx3000P Real-Time PCR apparatus (Agilent Technologies, CA, United States). In cases where a disagreement between results obtained from both technical duplicates was found, the sample assessment was repeated. Plasma levels for each miRNA were calculated in the form of a relative expression. This relative expression was calculated using the ΔCt method (i.e., the 2^-ΔCt^ algorithm was ΔCt = Ct_miR−x_ − Ct_miR−39_).

### 2.5. Objectives

Our objectives were to find relationships between selected miRNAs and the standard biomarkers of AMI as well as to find a panel of standard and potential biomarkers that might contribute to the identification of high-risk patients after acute myocardial infarction and post-pPCI treatment. The whole cohort was split according to the primary outcome (death within 365 days after AMI) into two subgroups (survivors and nonsurvivors), and both subgroups were characterized according to their biomarker levels.

### 2.6. Statistical Analysis

In this analysis, standard descriptive statistics were applied; absolute and relative frequencies were used for categorical variables and medians (supplemented with the 5th and 95th percentiles) were used for continuous variables (mean, SD, and CV were also used for the description of miRs). The statistical significance of differences among groups of patients was tested using Fisher's exact test for categorical variables and the Mann-Whitney test for continuous variables. The Spearman correlation coefficient was used for the analysis of the statistical relationship between miRNAs and the standard markers. Cut-off points (cut-off values) of predictors of all-cause death during 365 days were established by ROC analysis. The point that guarantees the greatest sum of sensitivity and specificity was chosen as the best point. Risk factors for all-cause death during 365 days were analyzed by a Cox regression model of proportional hazards. Analysis was performed in IBM SPSS Statistics 24.0 with 5% level of significance.

## 3. Results

### 3.1. Baseline Characteristics

The analysis involved 122 adult patients (78.7% men and 21.3% women) with AMI followed by pPCI; the median age was 61.1 years. All patients used either prasugrel (53.3%) or ticagrelor (46.7%) for antiplatelet therapy. The cohort of patients was split into two subgroups: nonsurvivors (*N* = 6) and survivors (*N* = 116). Only six patients died within one year after AMI (three patients from the prasugrel and three from the ticagrelor group): five died suddenly and one died while in the hospital from an unconfirmed diagnosis of pulmonary embolism. All patients in this subgroup had an LVEF ≥ 40% at their control visit, which was 2–3 months after discharge from the hospital. The baseline characteristics of all patients, and both subgroups, including their comparison, are shown in Table 1.

### 3.2. Correlation of miRNAs with Standard Biomarkers

The relative levels of all three miRNAs were related to the levels of standard biomarkers: hsTnT (at admission), hsTnT (24 hours after admission), NT-proBNP, GDF-15, cystatin C, and LVEF.

**Figure 1:**
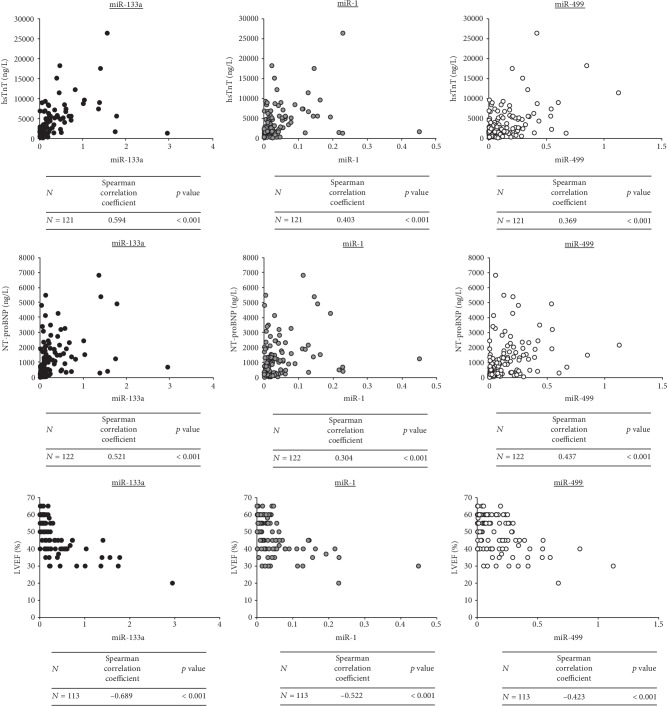
Correlations between particular miRNAs (relative expression) and hsTnT levels 24 hours after admission, NT-proBNP, and LVEF. hsTnT = high-sensitivity troponin T; LVEF = left ventricular ejection fraction; NT-proBNP = N-terminal prohormone of brain natriuretic peptide.

miR-133a and miR-1 weakly positively correlated with hsTnT at admission and strongly positively correlated with hsTnT 24 hours after admission (Figure 1). miR-499 moderately correlated with hsTnT 24 hours after admission. A strong negative correlation was found between all three miRNAs and the LVEF (Figure 1). A strong positive correlation was identified between both miR-133a and miR-499 and NT-proBNP, and a moderate positive correlation was found between miR-1 and NT-proBNP (Figure 1).

No correlation was found between any of the miRNAs and GDP-15 or cystatin C.

### 3.3. The Relationship between miRNAs and One-Year Mortality

The assessment of the prognostic potential of the selected biomarkers, for the identification of patients at increased risk of death, was based on their peripheral plasma levels and one-year survival.

**Figure 2:**
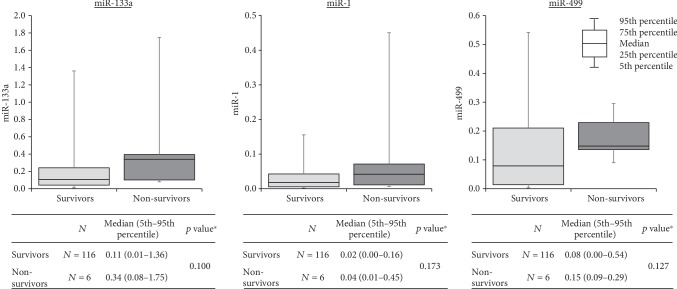
Relationship between miRNAs (relative expression) and one-year mortality. ^∗^Mann-Whitney test.

Median relative levels of miRNAs were higher in the nonsurvivor subgroup. But the total number of patients in this subgroup was small in comparison with that in the group of survivors (six vs. one hundred and sixteen), and the differences found did not reach statistical significance for any of the tested microRNAs (Figure 2).

The calculated cut-off values for miR-1, miR-133a, and miR-499 were 0.031, 0.330, and 0.088, respectively. Relative miRNA concentrations below these cut-off values were described as “low,” and those above the value were described as “high.”

Comparisons of the number of patients with low and high concentrations of particular miRNAs were made in both subgroups; in the nonsurvivor group, the relative frequency of high concentrations was higher, and in the case of miR-133a and miR-499, this difference reached statistical significance (Table 2, microRNAs). All 6 nonsurvivors had a high concentration of miR-499, whereas, in the survivor subgroup, only 46% of patients had a high concentration.

### 3.4. Relationship between Standard Biomarkers and One-Year Mortality

For the standard markers hsTnT and NT-proBNP, cut-off values were found in the same way as for miRNAs, and values were then described as either “low” or “high.” The cut-off value for hsTnT was 154.5 ng/L, and for NT-proBNP, it was 891.5 ng/L.

The number of patients with low and high concentrations of these two biomarkers was compared in both subgroups, and in the nonsurvivor group, the frequency of high marker levels was higher; in the case of NT-proBNP, this difference was statistically significant (Table 3, standard biomarkers). All patients who died within one year had a high concentration of NT-proBNP, whereas in the survivor group, only 43% had a high concentration of NT-proBNP.

### 3.5. Combinations of Biomarkers

Using the estimated cut-off values, two or three biomarkers were combined, in an effort to better describe the nonsurvivor subgroup and identify patients at risk of death. Combinations included (A) combinations of different microRNAs, (B) combinations of standard markers, and (C) combinations of microRNAs and standard markers. All tested combinations are shown in Table 3.

Based on a combination of NT-proBNP and miR-499 levels, a test group of 39 “at-risk” patients was created, which was 32% of the entire (survivor+nonsurvivor) cohort. The NT-proBNP and miR-499 combination criteria put all six nonsurvivors in the “at-risk” group, where they represented 15% of the “at-risk” group.

## 4. Discussion

In patients with a proven increased risk of death based on cardiovascular risk stratification during hospitalization, treatment with ACE inhibitors (or angiotensin AT1 blockers), beta-blocker therapy, and aldosterone antagonists are indicated when EF LK is ≤40% and/or there is heart failure [1]. Implantation of cardioverter-defibrillator (ICD) in a selected patient population is indicated when the indication criteria are met [1].

Despite the risk stratification of patients after myocardial infarction, ischemic complications recur even at low calculated risk, and these events can be fatal. miRNAs, as a group of the potential new markers, could help in the stratification of these patients. Then, if an increased miRNA value and usual risk parameters including LVEF are found without significant pathology, supplementation of the Holter ECG to exclude ventricular arrhythmias and careful follow-up of these patients should be considered.

For this reason, we used a well-defined and very homogeneous cohort of AMI patients after pPCI and tested the prognostic value of three cardiomyo-specific miRNAs (miR-1, miR-133, and miR-499) in one-year cardiovascular mortality and their relation to standard laboratory markers. We proved correlations between levels of miR-1, miR-133, and miR-499 with hsTnT, NT-proBNP, and LVEF in this cohort of patients. In addition, we found a possible relationship between combined levels of miR-499 with NT-proBNP and increased one-year mortality risk in these patients on dual antiplatelet therapy that has not been published yet.

### 4.1. MyomiR Levels after Myocardial Infarction

Many authors focus on miRNA levels during cardiovascular events and their possible contribution to the diagnostics or differential diagnostics [14, 18]. Published papers found that levels of miR-1 and miR-133a/b increase soon after AMI, reaching a peak shortly before TnI and returning to baseline within five days, while miR-499 peaks later, about 12 hours after the onset of the first symptoms [14]. miR-499 levels are naturally very low in healthy people and increase after AMI, with levels being higher in acute myocardial infarction with ST-segment elevation (STEMI) compared to non-STEMI patients [18], and provide a comparable diagnostic value to that of hsTnT [18]. Concentrations of miR-499 are higher in patients after AMI compared to patients with unstable angina [19]. miR-499 remains increased 24 hours after MI and then slowly decreases to original levels over 7 days [19]. Increased levels of circulating miR-499 and miR-208 after AMI reflect the cardiac damage caused by the AMI [19]. miR-208 levels are usually under the limits of detection in healthy individuals but rapidly increase after AMI. The peak is observed 3 hours after reperfusion, which is then followed by a rapid fall in concentration back to initial levels within 24 hours [20]. Since our samples were taken 24 hours after admission to the hospital, the concentration of miR-208 was either under the detection limit or too low to be quantified, so this miRNA was not included in our analysis, and only levels of miR-1, miR-133a, and miR-499 were measured.

### 4.2. Correlations of miRNAs with Standard Markers

We focused on the correlation with selected standard biomarkers and found a significant positive correlation of the three microRNAs with hsTnT and NT-proBNP. Our findings agree with other published papers, where levels of miR-499 were found to be positively correlated with levels of troponin T and I [14, 19, 21], despite minor differences in methods, the cohort of MI patients, and time of sampling. A positive correlation (*r* = 0.596, *p* < 0.001) between miR-133a and cTnI was previously published [19, 22] and reported a similar trend in levels of both markers in the early phase of AMI [22]; another work described an early miR-1, miRNA-133a, and miR-133b peak that occurred at a similar time as the TnI peak, whereas miR-499-5p exhibited a slower time course [14]. A correlation was also found between miR-499 and creatinine kinase (CK) [18, 19].

All the three analyzed miRNAs were found to have a moderate or strong positive correlation with NT-proBNP, which was published to be an important independent predictor of poor outcomes [23]. Furthermore, we found a strong negative correlation between all the three miRNAs and LVEF, which is in line with several other authors who found a similar negative correlation of miR-499 with LVEF (*r* = −0.36, *p* = 0.008) [16] or a weak negative correlation of miR-499-5p with LVEF (−0.11, *p* = 0.037) and miR-1 with LVEF (*r* = −0.16, *p* = 0.003) [21].

### 4.3. miRNAs in One-Year Prognosis

Finally, we looked for differences in the levels of laboratory markers between patients at an increased risk of death (nonsurvivors) and survivors. We found that all nonsurvivors had high levels of NT-proBNP and high levels of miR-499. Levels of NT-proBNP were measured in all AMI patients shortly after admission to the hospital before pPCI; in addition, the levels of microRNAs were also measured as potential new biomarkers. The choice of microRNAs was based on promising assessments for diagnostics or prognostics in recently published literature [14, 21, 22, 24].

Current risk stratification is based primarily on left ventricular dysfunction, measured as left ventricular ejection fraction [1, 2]. Many studies have found a clear relationship between reduced LVEF and mortality, which increases when LVEF falls under 50% and progressively increases when LVEF declines under 40% [2]. Despite this important predictor, about 50% of patients who die suddenly do not meet the abovementioned LVEF criteria [2]. Also, in our cohort, only 2 patients out of 6 in the nonsurvivor subgroup had an LVEF ≤ 35% during hospitalization and none at the time of follow-up. Our goal was to find a combination of laboratory markers that could contribute to the better identification of patients at increased risk of death after myocardial infarction and thus decrease the relatively high post-AMI mortality that reaches 7–20% at one year, 24–38% at five years, and 40–56% at ten years [2].

In our work, we analyzed cardio-enriched microRNAs, measurable 24 hours after patient admission to the hospital, to see if some of them could potentially fit into such a panel of biomarkers. Our results found that miR-499 in combination with NT-proBNP was best able to characterize the nonsurvivor subgroup. The number of papers dealing with myomiRs and AMI patients' prognosis is relatively limited. A recently published work confirms increased levels of cardio-enriched miRNAs (miR-499 and miR-208) in the blood of AMI patients and establishes an association of increased miRNA levels with reduced systolic function after AMI and risk of death or heart failure within 30 days [21]. Another work found that circulating levels of miR-133a and miR-208b were associated with all-cause mortality at 6 months, but this did not add prognostic information to hsTnT, the standard marker of AMI [25]. miR-133 was also studied in the high-risk STEMI patient cohort, where its levels provided prognostic information but do not add independent prognostic information to traditional markers of AMI [26].

In spite of the undeniable advantages of a well-defined and very homogeneous cohort of patients, this analysis was limited by the low number of patients in the nonsurvivor subgroup and by its retrospective character.

## 5. Conclusion

One-year mortality in patients after AMI treated with pPCI was very low (4.9%). A positive correlation was found between miRNA-1, miR-133a, and miR-499 and hsTnT (24 hours after admission) and NT-proBNP, and a negative correlation with LVEF. Further, this work suggests that plasma levels of circulating miR-499 might contribute to the identification of patients at increased risk of death, especially when combined with NT-proBNP levels. Further analyses are needed to determine if miR-499 or some other miRNAs can be effectively used in practice to better identify at-risk patients, to better understand the roles of these miRNAs in AMI, and to thus improve the clinical management of patients after AMI.

## Figures and Tables

**Table 1:** Baseline characteristics.

	All patients	Survivors	Nonsurvivors	*p* values
	Median (5th-95th percentile)			
Number of patients	122	116	6	
Age (years)	61.1 (40.4–76.8)	61.1 (40.1–76.7)	65.7 (56.1–81.0)	0.166
Men (number, %)	96 (78.7%)	91 (78.4%)	5 (83.3%)	0.999
BMI	27.6 (22.2–34.3)	27.6 (22.1–34.3)	26.7 (24.7–44.1)	0.929
Drug used: prasugrel (number, %)	65 (53.3%)	62 (53.4%)	3 (50.0%)	0.999
Drug used: ticagrelor (number, %)	57 (46.7%)	54 (46.6%)	3 (50.0%)	
STEMI (number, %)	121 (99.2%)	115 (99.1%)	6 (100.0%)	0.999
Left bundle branch block (LBBB) (number, %)	1 (0.8%)	1 (0.9%)	0 (0.0%)	0.999
Right bundle branch block (RBBB) (number, %)	1 (0.8%)	1 (0.9%)	0 (0.0%)	0.999
Hyperlipidaemia (number, %)	36 (29.5%)	35 (30.2%)	1 (16.7%)	0.669
Obesity (number, %)	23 (18.9%)	22 (19.0%)	1 (16.7%)	0.999
Arterial hypertension (number, %)	56 (45.9%)	52 (44.8%)	4 (66.7%)	0.412
Smoking (number, %)	84 (68.9%)	80 (69.0%)	4 (66.7%)	0.999
Diabetes mellitus (number, %)	17 (13.9%)	16 (13.8%)	1 (16.7%)	0.999
Time since the first symptoms to admission (hours)	3.0 (0.5–36.0)	3.0 (0.5–12.0)	6.0 (3.0–72.0)	0.061
Left ventricular ejection fraction (%)	50.0 (30.0–60.0)	55.0 (30.0–60.0)	45.0 (30.0–50.0)	0.054
Laboratory values (median (5th-95th percentile))				
hsTnT (at admission) (ng/L)	86.0 (12.0–1325.0)	84.0 (12.0–1325.0)	201.5 (27.0–4978.0)	0.257
hsTnT (24 hours after admission) (ng/L)	2432.0 (377.0–9651.0)	2324.0 (368.0–9651.0)	4306.5 (1526.0–15114.0)	0.201
Myoglobin (at admission) (*μ*g/L)	198.0 (30.0–1385.0)	176.0 (30.0–1547.0)	652.0 (161.0–1317.0)	0.066
Creatine kinase (at admission) (*μ*kat/L)	3.9 (1.4–23.7)	3.8 (1.3–23.7)	6.4 (2.6–26.8)	0.097
NT-proBNP (at admission) (ng/L)	757.0 (105.0–4142.0)	666.5 (104.0–4285.0)	1373.5 (904.0–3096.0)	0.074
Cystatin C (at admission) (mg/L)	121; 0.99 (0.80–1.47)	1.00 (0.79–1.49)	0.92 (0.85–1.09)	0.417
GDF-15 (at admission) (ng/L)	807.1 (372.8–1827.7)	796.3 (372.8–1827.7)	1044.9 (357.3–1848.8)	0.305

GDF-15 = growth/differentiation factor 15; hsTnT = high-sensitivity troponin T; NT-proBNP = N-terminal prohormone of brain natriuretic peptide; STEMI = acute myocardial infarction with ST-segment elevation.

**Table 2:** Relationship between individual marker levels and one-year mortality.

Marker	Gene	Concentration	Patients, number (%)	Survivors, number (%)	Nonsurvivors, number (%)	*p* value^∗^
	Locus (OMIM)					
MicroRNAs (relative concentration)						

miR-133a	MIR133A1/MIR133A2	<0.330	93 (76.2)	91 (78.4)	2 (33.3)	**0.028**
	18q11.2/20q13.33	≥0.330	29 (23.8)	25 (21.6)	4 (66.7)	

miR-1	MIR1-1/MIR1-2	<0.031	78 (63.9)	76 (65.5)	2 (33.3)	0.187
	20q13.33/18q11.2	≥0.031	44 (36.1)	40 (34.5)	4 (66.7)	

miR-499	MIR499	<0.088	63 (51.6)	63 (54.3)	0 (0.0)	**0.011**
	20q11.22	≥0.088	59 (48.4)	53 (45.7)	6 (100.0)	

Standard biomarkers (concentration in ng/L)						

hsTnT	TNN2	<154.5	77 (63.6)	75 (65.2)	2 (33.3)	0.189
	1q32.1	≥154.5	44 (36.4)	40 (34.8)	4 (66.7)	

NT-proBNP	NPPB	<891.5	66 (54.1)	66 (56.9)	0 (0.0)	**0.008**
	1p36.22	≥891.5	56 (45.9)	50 (43.1)	6 (100.0)	

^∗^Fisher exact test. NT-proBNP = N-terminal prohormone of brain natriuretic peptide; hsTnT = high-sensitivity troponin.

**Table 3:** Relationship between various combinations of marker levels and one-year mortality.

Markers and their levels	Patients, number (%)	Survivors, number (%)	Nonsurvivors, number (%)	*p* value^∗^
MicroRNAs				
miR-133a+miR-1				
Both low	75 (61.5)	73 (62.9)	2 (33.3)	**0.045**
One low and one high	21 (17.2)	21 (18.1)	0 (0.0)	
Both high	26 (21.3)	22 (19.0)	4 (66.7)	
miR-133a+miR-499				
Both low	57 (46.7)	57 (49.1)	0 (0.0)	**0.004**
One low and one high	42 (34.4)	40 (34.5)	2 (33.3)	
Both high	23 (18.9)	19 (16.4)	4 (66.7)	
miR-1+miR-499				
Both low	47 (38.5)	47 (40.5)	0 (0.0)	**0.019**
One low and one high	47 (38.5)	45 (38.8)	2 (33.3)	
Both high	28 (23.0)	24 (20.7)	4 (66.7)	
miR-133a+miR-1+miR-499				
All low	47 (38.5)	47 (40.5)	0 (0.0)	**0.003**
Minimum one low, minimum one high	55 (45.1)	53 (45.7)	2 (33.3)	
All high	20 (16.4)	16 (13.8)	4 (66.7)	

Standard biomarkers				
hsTnT+NT-proBNP				
Both low	42 (34.7)	42 (36.5)	0 (0.0)	**0.006**
One low and one high	58 (47.9)	56 (48.7)	2 (33.3)	
Both high	21 (17.4)	17 (14.8)	4 (66.7)	

MicroRNAs and standard biomarkers				
hsTnT+miR-133a				
Both low	63 (52.1)	62 (53.9)	1 (16.7)	**0.024**
One low and one high	43 (35.5)	41 (35.7)	2 (33.3)	
Both high	15 (12.4)	12 (10.4)	3 (50.0)	
hsTnT+miR-1				
Both low	53 (43.8)	52 (45.2)	1 (16.7)	0.094
One low and one high	48 (39.7)	46 (40.0)	2 (33.3)	
Both high	20 (16.5)	17 (14.8)	3 (50.0)	
hsTnT+miR-499				
Both low	39 (32.2)	39 (33.9)	0 (0.0)	**0.005**
One low and one high	61 (50.4)	59 (51.3)	2 (33.3)	
Both high	21 (17.4)	17 (14.8)	4 (66.7)	
NT-proBNP+miR-133a				
Both low	59 (48.4)	59 (50.9)	0 (0.0)	**0.003**
One low and one high	41 (33.6)	39 (33.6)	2 (33.3)	
Both high	22 (18.0)	18 (15.5)	4 (66.7)	
NT-proBNP+miR-1				
Both low	51 (41.8)	51 (44.0)	0 (0.0)	**0.014**
One low and one high	42 (34.4)	40 (34.5)	2 (33.3)	
Both high	29 (23.8)	25 (21.6)	4 (66.7)	
NT-proBNP+miR-499				
Both low	46 (37.7)	46 (39.7)	0 (0.0)	**0.001**
One low and one high	37 (30.3)	37 (31.9)	0 (0.0)	
Both high	39 (32.0)	33 (28.4)	6 (100.0)	

^∗^Fisher exact test: difference between both subgroups. hsTnT = high-sensitivity troponin T; NT-proBNP = N-terminal prohormone of brain natriuretic peptide.

## Data Availability

The data (miRNA Ct values and values of hsTnT and NT-proBNP) used to support the findings of this study are available from the corresponding author upon request.
